# Immunogenicity of Multiepitope Vaccine Candidate against *Toxoplasma gondii* Infection in BALB/c Mice

**Published:** 2018

**Authors:** Khalid HAJISSA, Robaiza ZAKARIA, Rapeah SUPPIAN, Zeehaida MOHAMED

**Affiliations:** 1. Dept. of Zoology, Faculty of Science and Technology, Omdurman Islamic University, Omdurman, Sudan; 2. Dept. of Medical Microbiology & Parasitology, School of Medical Sciences, Universiti Sains Malaysia, 16150 Ku-bang Kerian, Kelantan, Malaysia; 3. Biomedicine Program, School of Health Sciences, Universiti Sains Malaysia, 16150 Kuban Kerian, Kelantan, Malaysia

**Keywords:** *Toxoplasma gondii*, Multiepitope peptide, SAG1, GRA2, GRA7, Vaccine

## Abstract

**Background:**

*Toxoplasma gondii* is a widely prevalent intracellular protozoan parasite which causes serious clinical and veterinary problems. Development of an effective vaccine for controlling toxoplasmosis is an extremely important aim. In the present study, the protective efficacy of recombinant multiepitope antigen (USM.TOXO1) expressing nine potential epitopes identified from SAG1, GRA2, and GRA7 of *Toxoplasma gondii* was evaluated in BALB/c mice.

**Methods:**

Mice were immunized subcutaneously with three doses of USM.TOXO1 antigen (10 μg/ml). Following the immunization, the IgG antibody, IgG subclass, IFN-γ and IL-4 production were evaluated using ELISA, the study was conducted at Animal Research and Service Center (ARASC), USM Health Campus in 2016.

**Results:**

Mice immunized with USM.TOXO1 significantly induced a mixed Th1/Th2 response polarized toward the IgG1 antibody isotype. While the cytokine analysis revealed a significant release of IFN-γ cytokines.

**Conclusion:**

USM.TOXO1 is a potential vaccine candidate that elicits strong immunity in BALB/c mice. The proven immunogenicity of the generated antigen can serve as a premise for further use of epitope-based vaccine in the immunoprevention of human and animal toxoplasmosis.

## Introduction

Toxoplasmosis caused by the obligate intracellular protozoan parasite *Toxoplasma gondii*, is a widespread zoonotic disease with significant medical and veterinary importance (1, 2). Controlling the lethal impact of the disease is currently depends on chemotherapy to completely prevent or cure toxoplasmosis in humans (3). However, chemotherapy provides limited results, and several drugs could cause severe side effects (4). Under this scenario, developing effective and safe vaccines is the need of the hour because it becomes the appropriate way to prevent the diseases (5).

Currently, vaccination is considered as a highly effective strategy for disease prevention. The efficacy of vaccination in controlling various infectious diseases, including toxoplasmosis, has been extensively studied and verified (5). Consequently, the vaccination approaches for *T. gondii* infection in the last 20 years were based on different types of immunogens, including live-attenuated parasites, killed vaccines, native parasite antigens, DNA vaccines and recombinant antigens (6–8).

Developing of potential *T. gondii* vaccines possessing high immunogenic characteristics is a challenging research goal (9). An ideal vaccine against toxoplasmosis should express different stages of the parasite life cycle to allow the induction of a strong and broad immune response (10). Regardless of the intensive efforts and significant advances in the development of an effective *T. gondii* vaccine, no protective vaccine for human use has yet been achieved (5). Meanwhile, the already approved animal vaccine has shown limited efficacy (11). The major challenge is the lack of efficacious antigen candidates because of the complex *T. gondii* life cycle. Nevertheless, various candidate antigens have been identified, and those that are able to induce a strong and long-lasting immunity are limited.

Recently, epitope-based vaccines have attracted considerable attention as a potential means for promoting protective immune responses against *T. gondii* infection (12, 13). The application of such vaccine offers potential advantages, such as the ability to enhance a specific immunity against the selected epitopes, the possibility to induce variety of immune response types, increased safety and the opportunity to engineer the epitopes (14).

Accordingly, considerable efforts have been made to develop an epitope-based vaccine against *T. gondii* infection. Indeed, epitope-based vaccines induce protective immune response against the *T. gondii* parasite (3, 12). The variability of *T. gondii* antigens across the complicated life cycle of the parasite renders the use of multiepitope vaccines as a promising immunization strategy against toxoplasmosis (10).

In this study, a synthetic multiepitope antigen named as USM.TOXO1 expressing nine potential B cell epitopes identified from SAG1, GRA2, and GRA7 of *T. gondii* was developed. Consequently, the humoral and cellular immune responses elicited by this vaccine in BALB/c mice were evaluated.

## Materials and Methods

### Epitopes prediction

The immunodominant B cell epitopes expressed within the SAG1, GRA2 and GRA7 of *T. gondii* were predicted by the ABCpred online prediction server (15). Following the prediction, and based on the immunogenicity score three epitopes from each protein (16 amino acids in length each) were selected as potential epitope candidates in the choice of the synthetic gene construction.

### Construction of USM.TOXO1 synthetic gene

The DNA sequences of the identified epitopes were retrieved from the gene bank and used to design a single synthetic gene encoding all selected epitopes with a final length of 435 bp, expected to express the most reactive epitopes within SAG1, GRA2 and GRA7 antigens. Consequently, the gene was constructed by assembly PCR (16). Subsequently, the gene was cloned into pET32a vector.

### Expression and purification of the recombinant multiepitope antigen

*E. coli* BL21 (DE3) pLyS cells containing pET-32a.rMEP were grown in Luria–Bertani (LB) broth, supplemented with 100 μg/ml ampicillin with various shaking (225 rpm) at 37 °C until the optical density (OD) at 600 nm reaches 0.4–0.6. The protein expression was then induced by isopropyl-D thiogalactopyranoside (IPTG) with final concentration 1 mM. The synthetic protein was purified using Ni-NTA column according to the manufacturer’s instruction.

### Mice and ethics statement

Fourteen male BALB/c mice (8–12 wk old) purchased from Animal Research and Service Center (ARASC), USM Health Campus in 2016, were used for the experiments. The approval for all experimental procedures was obtained from the Animal Ethics Committee, Universiti Sains Malaysia (approval No. 2015 (95) (608)).

### Mouse immunizations

The immunization experiment was carried out as described previously (17). Briefly, seven mice (immunized group) were subcutaneously immunized three times with 10 μg/ml of USM.TOXO1 antigen emulsified with Freund’s complete adjuvant in the first dose, or Freund’s incomplete adjuvant in the second and third doses, at two weeks intervals. While phosphate-buffered saline emulsified with appropriate adjuvant was similarly injected to another group of mice (control group). The mice were daily monitored to ensure that there were no adverse effects of the vaccine.

### Measurement of humoral response

Collected mice sera were used to quantify the presence of anti-USM.TOXO1 specific IgG and IgG subclass antibodies using ELISA. Briefly, the purified USM.TOXO1 was diluted in 0.05 M carbonate buffer (pH 9.6) to the final concentration of 2.5 μg/ml. A volume of 100 μl of this dilution was then added to each well of a 96-well microplate and incubated at 4 °C overnight. Following the incubation, the plate was then washed three times with PBS-T for 5 min and blocked with PBS supplemented with 3% of bovine serum albumin (blocking buffer) at 37 °C for 1 h. The washing step was repeated three times. Subsequently, 100 μl of sera from immunized and control mice diluted 1:400 in blocking buffer was dispensed into each well and incubated for 30 min at 37 °C. The wells were again washed prior to the addition of 100 μl of HRP conjugated anti-mouse IgG, IgG1, IgG2a and IgG2b antibodies (Abcam, USA) at a 1:10000 dilution in PBS and the plate was incubated at 37 °C for 30 min. Another three rounds of washes were carried out and the color reaction was allowed to develop by adding 100 μl of TMB substrate followed by 15 min incubation. Consequently, the color development was stopped by the addition of 100 μl of 2 M H_2_SO_4_ and the optical density at 450 nm was determined on microplate readers.

### In vitro production of cytokines from splenocytes after USM.TOXO1 stimulation

The levels of cytokines production were determined using splenocytes from three mice per group two weeks after the final immunization. The splenocytes were cultured with USM.TOXO1 (10 μg/mL) in 96-well plates at 37 °C in 5% CO_2_ (18). Parallelly, Concanavalin A and PBS were used as positive and negative controls respectively. The cells were harvested and the supernatants were assessed for the secretion of interleukin-4 (IL-4) at 24 h and interferon gamma (IFN-γ) at 72 h, using commercial ELISA kit (Thermo Scientific, USA) according to the manufacturer’s instructions.

### Statistical analysis

The statistical analysis between the two groups was performed using SPSS software (Chicago, IL, USA). The levels of antibodies and cytokines production in the immunized and control groups of BALB/c were analyzed by the U Mann–Whitney test. Differences were considered to be significant with *P*<0.05.

## Results

### Production of the USM.TOXO1 multi-epitope antigen

A single synthetic gene encoding the most reactive epitopes within SAG1, GRA2, and GRA7 antigens was constructed using assembly PCR. The corresponding DNA was successfully used to express a novel multiepitope USM.TOXO1 antigen in *E. coli* system and purified as previously described (16).

### Antibody responses in immunized BALB/c mice

**Fig. 1:**
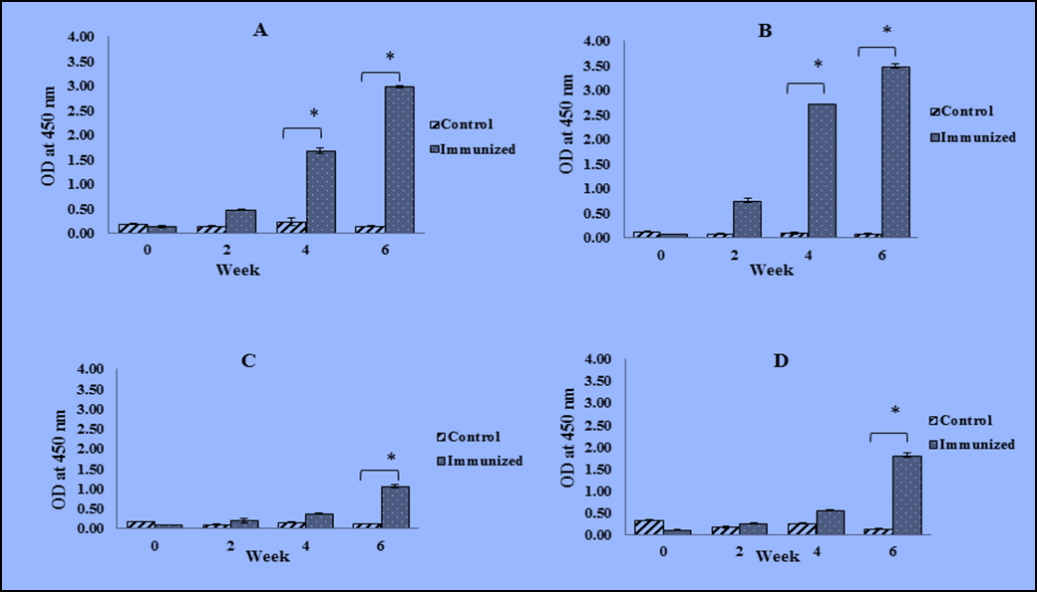
Serum levels of total IgG (A), IgG1 (B), IgG2a (C) and IgG2b (D) antibodies (OD_450_ ± SD) in immunized and control groups of mice, at week 0, 2, 4, and 6. Significant differences between control and immunized groups are marked with (*), where *P < 0.05

The titers of total IgG antibodies, besides the IgG isotypes (IgG1, IgG2a, and IgG2b) induced by the USM.TOXO1 in immunized mice at weeks 0, 2, 4 and 6 post immunization were measured by using ELISA. High levels of specific anti-USM.TOXO1 IgG antibodies were detected in immunized mice following booster immunization, especially on the fourth and sixth weeks after the first immunization (P < 0.05) (Fig. 1 A), However, analysis of IgG production in control mice showed no increase in the IgG titers following booster immunizations.

The recombinant USM.TOXO1 antigen-stimulated a high IgG1 response two weeks after the first immunization. The antibody response was continuously increased after the second and third booster immunization, in which the IgG1 titer was statistically significant in the immunized mice versus control group (*P*<0.05) (Fig. 1B).

In contrast, IgG2a isotype showed a slight increase in the antigen-specific IgG2a levels after the first and second immunization. However, a significant increase in anti-USM.TOXO1 IgG2a antibody titers were observed in immunized mice sera (*P*<0.05) two weeks after the final booster immunization. While in control group, the level of IgG2a antibodies remained low throughout the immunization period and was equivalent to the level prior to immunization. The USM.TOXO1 has the potential to induce mixed Th1 and Th2 response. However, the antibody pattern predominantly exhibited a Th2-type response. Additionally, significant increase in the IgG2b level was also observed in the immunized group compared to the control group (Fig. 1 D).

### In vitro production of cytokines from splenocytes after USM.TOXO1 stimulation

**Fig. 2:**
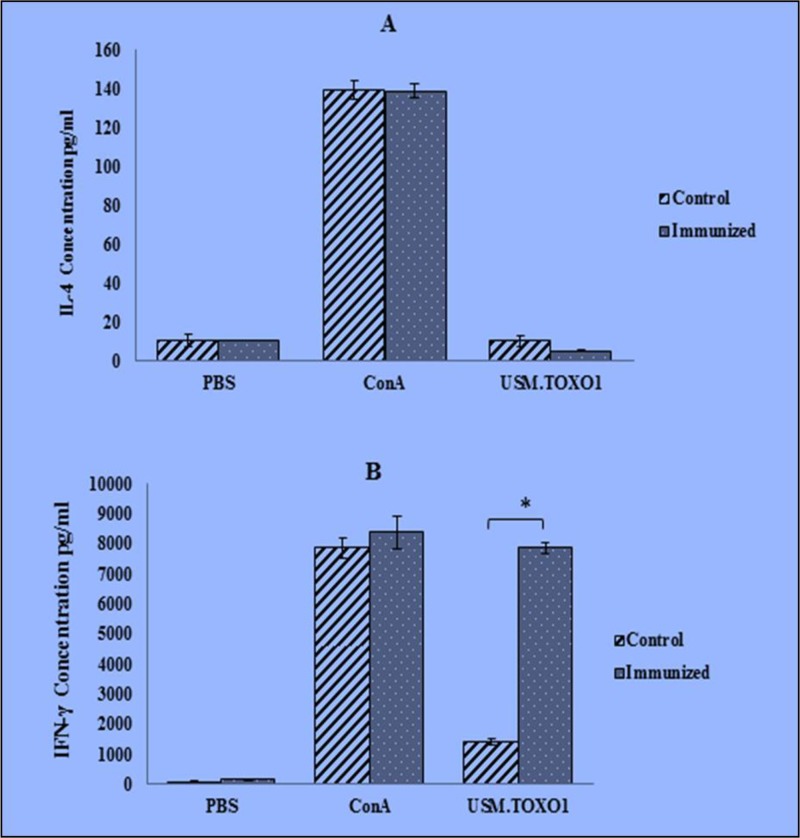
IL-4 (A) and IFN-γ (B) production by splenocytes isolated from USM.TOXO1-vaccinated and nonvaccinated mice BALB/c mice stimulated in vitro with USM.TOXO1, PBS or ConA. Antigen-induced cytokines release was determined after 24 h (IL-4) or 72 h (IFN-γ) of stimulation by ELISA. The concentration of IL-4 and IFN-γ in experimental and control samples was calculated on the basis of the respective standard curves. Results are representative of one of three similar experiments. Data are expressed as mean ±SD. * *P* < 0.05

A significant level of IFN-γ (fivefold) was released in the supernatants of the restimulated splenocyte culture from USM.TOXO1 immunization mice compared with the control group (Fig. 2). On the other hands, there was no significant evidence of released IL-4 in the splenocyte cultures from both immunized and control mice. Mice immunization with USM.TOXO1 antigen did not enhance the *in vitro* release of IL-4 following the stimulation.

## Discussion

Development of effective vaccines against *T. gondii* parasite may contribute significantly in preventing and controlling the spread of the disease, which is important for the improvement of toxoplasmosis outcome in both humans and livestock animals (5). In this study, a multiepitope-based vaccine expressing nine potential immunodominant epitopes identified from SAG1, GRA2, and GRA7 was evaluated as potential *T. gondii* vaccine. The rational selection of the *T. gondii* antigens that possess conserved T and B cell epitopes is crucial for the successful application of this epitope-based strategy (19). Thus, SAG1, GRA2, and GRA7 have been selected as the candidate antigens to be assessed in the current project. All of these antigens have been the subject of various fundamental studies. The findings of most of these studies demonstrated the potential of these antigens to become more successful diagnostic reagents or/and effective vaccines.

SAG1 is of particular interest because it represents around 5% of the tachyzoite antigen (20). Investigations on the immunogenicity and immunoreactivity of SAG1 repeatedly yielded significant results (21, 22). These reasons explain the selection of SAG1 as an antigen candidate in this study. GRA7 is a promising vaccine candidate and novel diagnostic reagent (23). Direct contact of GRA7 with the host immune system enhances the induction of strong antibody and cell-mediated responses in both acute and chronic infection (24).

Similar to SAG1 and GRA7, GRA2 is also characterized as a highly immunogenic antigen during *T. gondii* infections; it has the potential to induce protective immune response in both human and experimental models (25). SAG1, GRA7, and GRA2 antigens could advance the development of effective diagnostic reagents for *T. gondii*.

USM.TOXO1 vaccine was able to elicit strong humoral and cellular immune responses. Significantly high levels of total IgG antibodies were observed in the sera of USM.TOXO1-immunized mice compared to the PBS-immunized mice. In particular, the level of USM.TOXO1-specific IgG antibodies in the sera obtained from vaccinated mice gradually increased with booster immunization, especially on the fourth and sixth weeks after the first immunization (*P*<0.05). However, the IgG levels did not increase and remained low throughout the experiment in the control mice. There was the substantial role of the IgG antibodies in the protection against *T. gondii* infection (20, 26). The production of anti-*T. gondii* IgG antibodies enable parasite destruction via various mechanisms, including activation of the complement system, phagocytosis, and blocking the parasite receptor. These strategies were found to be directly correlated with protection against toxoplasmosis (27, 28). Thus, the IgG response is considered as an important component of immunization against *T. gondii*.

Immunization of BALB/c mice with USM.TOXO1 led to the production of high anti-USM.TOXO1 IgG1 levels. By contrast, a significant increase in anti-USM.TOX1 IgG2a antibody was also observed in the immunized mice sera. Similarly, the immunized mice generated high levels of IgG2b than those injected with PBS (*P*<0.05). Mice immunization with USM.TOXO1 generated a mixed Th1/Th2 response, with a predominant synthesis of IgG1 isotype after the booster immunization. Interestingly, USM.TOXO1 is a promising vaccine; this finding is supported by the assumption that a good vaccine should induce both cellular Th1 and humoral Th2 responses (29).

Similarly, induction of protective immunity accompanied by the production of antigen-specific IgG1 and IgG2a antibody subclasses was previously observed. Mice immunization with recombinant antigens resulted in the induction of a mixed Th1/Th2 antibody response, with the predominance of the Th2 type (26). Conversely, the predominance of Th1 immune response is frequently reported as a commonly observed phenomenon during the evaluation of several *T. gondii* vaccines (23, 30).

Th1-biased immune response is known as an effective immunological mechanism required in limiting parasite spread, reduce the brain cyst formation, and prolong the survival time of the immunized mice after challenge with a lethal dose of tachyzoites (31, 32). However, prolonged protection and increase in survival rate, mediated by the Th2 immune response have also been reported (33). Moreover, the type of immunity has also been correlated with the genetic background of the mice and the type of adjuvant used (34). Therefore, directing the immune response generated by USM.TOXO1 towards the Th1 type would be a very promising and valuable strategy. This manipulation can be achieved by using appropriate adjuvants (35). Besides, the capability of the mixed Th1/Th2 response generated by the USM.TOXO1 antigen in protecting the mice and increasing the survival rate after parasite challenge was not examined in this study and hence requires further investigation.

Another important strategy in the resistance against the *T. gondii* parasite is cellular immunity activation. This kind of immunity is the key mechanism in developing effective host protection during *T. gondii* invasion (36). The effectiveness of cellular immunity against *T. gondii* infection has been suggested to be mainly due to the potential role of IFN-γ in parasite clearance (29). Therefore, an appropriate vaccination strategy should promote significant Th1 response marked by elevated IFN-γ production (12).

Splenocytes from both immunized and control mice were stimulated with USM.TOXO1 antigen in vitro. There was a significantly higher IFN-γ level in the immunized mice than in the control mice (Fig. 2). However, immunization with the USM.TOXO1 antigen did not increase the in vitro release of IL-4 after splenocyte stimulation with the purified antigen. The cellular immunity induced by USM.TOXO1 is a Th1 type response. However, many factors can influence the differentiation of the Th cells into Th1 or/and Th2. For example, IL-12 and IL-18 enhance the Th1-type immune responses, whereas IL-5 and IL-6 promote the Th-2 type immune responses. In this study, these factors were not explored.

Compared with humoral immunity, a slight variation in cellular immunity was detected. The USM.TOXO1 elicited a mixed Th1 and Th2-type immune response, with the predominance of IgG1 antibody isotypes. Meanwhile, the cytokine analysis following the in vitro stimulation indicated a strong Th1 immune response. The immunization of BALB/c mice with multi-antigenic vaccine induces protective immunity accompanied by the production of IgG1 and IgG2a antibody responses with in vitro synthesis of IFN-γ cytokines (37).

Vaccination with *Toxoplasma* lysate antigen (TLA) induced effective humoral and cell-mediated immune responses, marked with high levels of toxoplasma-specific IgG1, IgG2a, and IFN-γ (10). The generated immunity significantly reduced the number of brain cysts in vaccinated mice. TLA is a mixture of diverse *T. gondii* antigens; the protective immunity induced in immunized mice is probably due to the potential property of TLA to activate both innate and adaptive immune responses. Immunization with a multi-antigenic vaccine expressing different stages of the parasite life cycle was shown to induce a broader and longer lasting protection (10). The finding strongly supported the result obtained after immunization with the USM.TOXO1 antigen, as USM.TOXO1 also express multi-antigenic determinant of *T. gondii* parasite.

For vaccines to be effective against *T. gondii* they should induce protective cellular and humoral responses (38). The increased expression of IFN-γ and the enhanced antibody response reported in this study indicated the induction of an efficient immune response against the USM.TOXO1 antigen. The question lies on whether the induced mixed immune response can provide protective immunity against *T. gondii* infection after parasitic challenge. The presented results also suggested that the generated multiepitope antigen offers the potential as a viable vaccine candidate. Moreover, challenge experiments are needed to verify the protective ability of the induced immune response.

## Conclusion

In the BALB/c model, vaccination with the USM.TOXO1 is sufficiently potent to elicit significant humoral and cellular immune responses. The strategy of using multi-epitope antigens seems to be highly promising in the development of potential vaccine candidates that would generate lasting protective immune responses against *T. gondii*. Furthermore, the use of epitope-based vaccine could be an important approach in investigating the improvement of the vaccination strategy in the future.
